# Modified Landauer Principle According to Tsallis Entropy

**DOI:** 10.3390/e26110931

**Published:** 2024-10-31

**Authors:** Luis Herrera

**Affiliations:** Instituto Universitario de Física Fundamental y Matematicas, Universidad de Salamanca, 37007 Salamanca, Spain; lherrera@usal.es

**Keywords:** Landauer principle, information theory, general relativity, gravitational radiation

## Abstract

The Landauer principle establishes a lower bound in the amount of energy that should be dissipated in the erasure of one bit of information. The specific value of this dissipated energy is tightly related to the definition of entropy. In this article, we present a generalization of the Landauer principle based on the Tsallis entropy. Some consequences resulting from such a generalization are discussed. These consequences include the modification to the mass ascribed to one bit of information, the generalization of the Landauer principle to the case when the system is embedded in a gravitational field, and the number of bits radiated in the emission of gravitational waves.

## 1. Introduction

The Landauer principle ref. [1], which is a cornerstone in the theory of information, states that the erasure of one bit of information stored in a system implies the dissipation of energy, whose value cannot be smaller than

(1) ΔE=kTln2,

where *k* is the Boltzmann constant and *T* denotes the temperature of the environment. The important point to keep in mind here is that even though the value of dissipated energy depends on the erasure procedure, it cannot be lower that Equation (1).

At this point, some remarks are needed, as follows:

- It has been argued in the past (see for example ref. [2]) ) that the main idea stated in the Landauer principle appears already in some Brillouin works ref. [3]. We shall skip this controversy and shall adopt the notation used by most of researchers, and we shall refer to it as the Landauer principle.
- In spite of some arguments put forward in the past questioning the relevance of the Landauer principle (see ref. [4] and references therein), the important point to retain here is that on the one hand it allows an “informational” reformulation of thermodynamics, as stressed in ref. [4], and on the other hand brings out a link between information theory and different branches of science refs. [5,6,7]. This allows us to approach some physical problems from the point of view of information theory.
- The expression Equation (1) for the dissipated energy heavily relies on the concept of entropy. More specifically, such an expression was found using the Gibbs entropy. Therefore, we should expect different expressions for alternative definitions of entropy.

## 2. Landauer Principle and Definition of Entropy

In order to exhibit the link between the Landauer principle and the definition of entropy, let us present a very simple proof of this principle.

Thus, let us consider a physical system which may be in two possible states, e.g., a particle whose spin may point upward or downward. The particle is inside a black box, and for an observer outside the box, the particle may be in either state with the same probability. Then, using the Gibbs definition of entropy given by

(2) $$S=-k\sum_{i=1}^{N}p_{i}\ln p_{i},$$

where *N* denotes the total number of accessible states and $p_{i}$ is the probability of each state (i.e., $\sum_{i=1}^{N}p_{i}=1$), we find that the Gibbs entropy of our system is

(3) S=kln2.

Let us now apply a magnetic field to our system as a consequence of which the spin is set to point to a determined direction (upward or downward). Obviously after such operation the entropy of the system becomes equal to zero, implying that there has been a decreasing of entropy equal to

(4) ΔS=kln2,

which according to the second law of thermodynamics should be accompanied by an increasing of, at least, the same amount, producing a dissipation of energy equals to

(5) ΔE=kTln2,

where *T* is the temperature of the environment.

Now, applying a magnetic field to our system, we set the direction of the spin in a predetermined direction, thereby erasing the information about where the spin was pointed to before switching on the magnetic field ref. [8]. Since this information is contained in the answer to the single question, “where is the spin pointing to?”, the amount of this information is one bit.

Thus, we have proved that erasing one bit of information implies that an amount of energy not smaller than Equation (5) must be dissipated, which is just the statement of the Landauer principle. The purpose of the above exercise being to bring out the relationship between the minimal amount of dissipated energy with the definition of entropy Equation (2).

Arriving at this point the obvious question arises: what could be the corresponding minimal amount of dissipated energy in the process of erasure of one bit of information if we resort to a definition of entropy different from Equation (2)?

We endeavor in this work to answer to the above question in the case when we use the Tsallis entropy (instead of using Equation (2)).

However, it would be legitimate to ask why, in particular, we have chosen Tsallis entropy, instead of any other definition of entropy? The answer to this question is based on the great deal of attention received by Tsallis proposal and its applications (see for example refs. [9,10,11,12,13] and references therein). Nevertheless, it goes without saying, that resorting to any other alternative definition of entropy would also deserve to be considered.

## 3. Tsallis Entropy and Modified Landauer Principle

Some years ago Tsallis proposed a generalization of Gibbs definition of entropy, which reads ref. [14]

(6) $$S=k\frac{1-\sum_{i=1}^{N}p_{i}^{q}}{q-1},$$

where *q* is a real number.

It is a simple matter to check that

(7) $$\lim_{q\to 1}k\frac{1-\sum_{i=1}^{N}p_{i}^{q}}{q-1}=-k\sum_{i=1}^{N}p_{i}\ln p_{i}.$$

Thus, deviations from the Gibbs entropy correspond to values of *q* different from 1.

Since its publication the Tsallis proposal has received a great deal of attention, and therefore we find it useful to evaluate its impact in the Landauer principle.

### The Lower Bound of the Dissipated Energy Ensuing the Erasure of One Bit of Information According to the Tsallis Entropy

In order to calculate the minimal amount of energy that must be dissipated when erasing on bit of information according to Tsallis entropy, let us retrace the steps of the exercise proposed in the previous section, leading to Equations (4) and (5).

Thus, let us consider a system with two possible accessible states (N=2) the probability of each of which is 1/2. Then, it follows from Equation (6) that the Tsallis entropy of our system is given by

(8) $$S=\frac{k}{q-1}\left[1-2^{\left(1-q\right)}\right].$$

We now proceed to apply a magnetic field to our system, after which the system is in a single state with probability 1, implying the erasure of one bit of information, and the vanishing of the entropy. Thus, the decreasing of entropy is given by

(9) $$\Delta S=\frac{k}{q-1}\left[1-2^{\left(1-q\right)}\right],$$

producing an amount of dissipated energy equal to

(10) $$\Delta E\equiv T\Delta S=\frac{kT}{q-1}\left[1-2^{\left(1-q\right)}\right].$$

**Figure 1:**
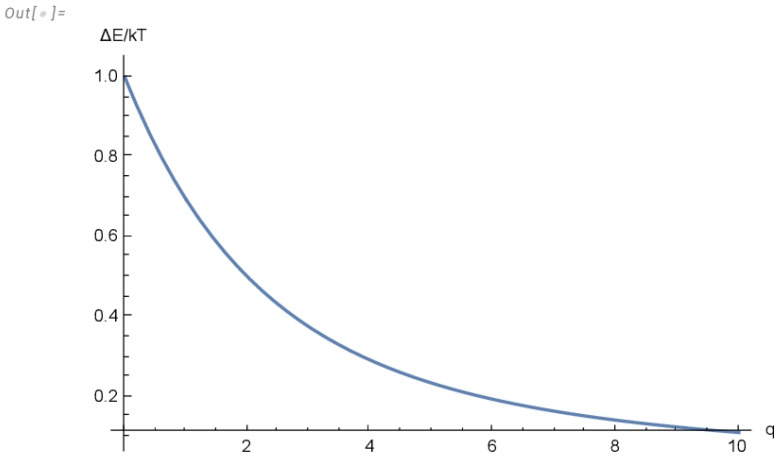
ΔE/kT as function of *q* for the Tsallis entropy.

As depicted in Figure 1, the above expression decreases monotonically with *q* for any q>0. It is a simple matter to check that in the limit q→1, expressions Equations (9) and (10) become Equations (4) and (5), respectively.

Thus, using the Tsallis entropy we see that the minimal energy dissipated in the erasure of one bit of information depends on the parameter *q* as expressed by (10).

We shall next see how this change affects some consequences derived from the Landauer principle.

## 4. The Mass of a Bit of Information

As we have seen above, the Landauer principle based on the Gibbs definition of entropy, asserts that a minimal amount of energy given by Equation (1), should be dissipated when erasing one bit of information. This fact implies the association of such an amount of energy with one bit of information. From the previous comment it follows at once, recalling the well-known fact that a mass $E/c^{2}$ has to be ascribed to energy *E*, and that a mass should be ascribed to a bit of information ref. [15] (see also refs. [4,16]), specifically

(11) $$\Delta M=\frac{kT}{c^{2}}\ln2,$$

where *c* denotes the speed of light.

Following the same reasoning leading to Equation (11), but using Equation (10) instead of Equation (1) we obtain for the mass associated with a bit of in formation

(12) $$\Delta M=\frac{kT}{c^{2}\left(q-1\right)}\left[1-2^{\left(1-q\right)}\right],$$

which of course becomes Equation (11) in the limit q→1.

From the above it follows that for one bit of information, at room temperature, the minimal dissipated energy is

(13) $$\Delta E\approx \frac{4.04}{q-1}\left[1-2^{\left(1-q\right)}\right]\times 10^{-14}erg$$

and the associated mass is:

(14) $$\Delta M\approx \frac{4.33}{\left(q-1\right)}\left[1-2^{\left(1-q\right)}\right]\times 10^{-35}g.$$

In the limit q→1, the above expressions yield $2.8\times 10^{-14}erg$ and $3\times 10^{-35}g$, respectively.

Also it is worth noticing that according to the uncertainty principle, there is a minimal time interval required to measure a given amount of energy. In our case this implies that for the energy Equation (13) the minimal time interval is

(15) $$\Delta t\approx \frac{\hbar }{\Delta E}\approx \frac{2.56(q-1)}{1-2^{1-q}}\times 10^{-14}s,$$

where *ℏ* is the Planck constant divided by 2π, thereby imposing a limit in the speed of information processing which in the case q≈1 is $\approx 10^{5}\;\mathrm{GHz}$.

We shall next consider the case, when the system is placed in a gravitational field.

## 5. Landauer Principle in a Gravitational Field

If the system is located in a (weak) static gravitational field, then the gravitational potential affects the Landauer principle. This important result was obtained by Daffertshoffer and Plastino ref. [17]. More specifically, these authors show that in this case (assuming for the entropy the Gibbs definition Equation (2)) the minimal amount of energy dissipated in the erasure of one bit of information is given by

(16) $$\Delta E=kT(1+\frac{\phi }{c^{2}})\ln2.$$

where ϕ denotes the (negative) gravitational potential, and $T(1+\frac{\phi }{c^{2}})$ (the Tolman’s temperature) is the quantity which is constant in thermodynamic equilibrium ref. [18].

Now, Equation (16) was obtained in the context of Newtonian gravity (weak field approximation). The extension of the above result to the general relativistic case is simple to achieve if we recall that in such a case Tolman’s temperature becomes $T\sqrt{g_{tt}}$, where $g_{tt}$ denotes the tt component of the metric tensor (the coefficient of $dt^{2}$ in the expression for the line element). Therefore, Equation (16) generalizes to

(17) $$\Delta E=kT\sqrt{g_{tt}}\ln2,$$

producing for the mass ascribed to a bit of information

(18) $$\Delta M=\frac{kT}{c^{2}}\sqrt{g_{tt}}\ln2.$$

So, the question is: how does the Landauer principle change in the presence of a gravitational field if we use the Tsallis entropy Equation (6) instead of Equation (2)?

Retracing the same steps followed in ref. [17], we obtain for the energy dissipated in the erasure of one bit of information and for the corresponding mass

(19) $$\Delta E=\frac{kT}{q-1}\left[1-2^{\left(1-q\right)}\right]\left(1+\frac{\phi }{c^{2}}\right),$$

and

(20) $$\Delta M=\frac{kT}{c^{2}\left(q-1\right)}\left[1-2^{\left(1-q\right)}\right]\left(1+\frac{\phi }{c^{2}}\right).$$

The generalization of the above expressions to the general relativistic case may be easily obtained by replacing $\left(1+\frac{\phi }{c^{2}}\right)$ with $\sqrt{g_{tt}}$.

After the formation of a black hole ($g_{tt}=0$), it follows from Equation (17) or Equation (19) that the energy dissipated during the erasure of one bit of information vanishes (assuming that the proper temperature is not singular), leading to a vanishing mass for a bit of information.

Now, the change of information without dissipation implies that all bits are already in one state only ref. [8]. This result agrees with the well-known assumption that the quantum radiation emitted by the black hole is nearly thermal (i.e., it conveys no information) refs. [19,20], thereby suggesting the “bleaching” of information at the horizon.

Thus, the well-known fact that after the formation of the horizon ($g_{tt}=0$), no further information leaves the system, follows in a simple way from information theory.

If the gravitational field does not correspond to a black hole ($g_{tt}\neq 0$), then we see a decreasing of the corresponding mass of a bit of information. Such a decreasing value depending on the parameter *q* occurs if we assume the Tsallis definition of entropy. At any rate such a decreasing value is very small for a weak gravitational field ($\frac{\phi }{c^{2}}\approx 10^{-9}$ for the case of the earth).

## 6. Gravitational Radiation, Radiated Information, and the Landauer Principle

Finally, we would like to consider the relationship between the energy and the information conveyed by gravitational radiation and the definition of entropy.

As we know from field theory (at classical level and for any spin), information about changes in the structure and/or state of motion of the source is propagated by radiation. Once the observers have received this information, the information encrypted in the “old” multipole structure is erased. In other words, the process of radiation implies not only propagation of information but also erasure of information, from which it is obvious that the Landauer principle should be implicated in the whole process.

The above comments imply that according to the Landauer principle, gravitational radiation entails a dissipation of energy. This conclusion was proved to be true in ref. [21] and is a consequence of the fact that gravitational radiation is an irreversible process, and this irreversibility should show up in the equation of state of the source.

This “informational” approach to radiation is particularly manifest in the Bondi formalism refs. [22,23].

In this approach there is a function (called “news function” by Bondi), which entails all the information required to forecast the evolution of the system (besides the initial data) and is identified with gravitational radiation itself. Such an identification is possible because the news function describe all changes in the field produced by changes in the source. Moreover, the vanishing of the news function is the necessary and sufficient condition for the total energy of the system to be constant. This scheme applies to Maxwell systems in Minkowski spacetime ref. [24] as well as to Einstein–Maxwell systems ref. [25].

Once we admit that a bit of radiated information implies a bit of erased information at the radiating system, which in turn leads to a decreasing of its total mass (energy), then it is legitimate to ask: what part of the total radiated energy (mass) corresponds to the radiated information?

We shall answer to this question, adopting the Tsallis definition of entropy.

In ref. [26] an answer was provided to the above question, based in the Landauer principle expressed through the Gibbs entropy Equation (1).

Thus, one obtains for the total dissipated energy (see ref. [26] for details)

(21) $$E_{rad}^{\left(L\right)}=\int_{r_{\Sigma }}^{\infty }\int_{0}^{\pi }\int_{0}^{2\pi }\sqrt{\left|g\right|}\mu_{rad}^{\left(L\right)}drd\theta d\phi ,$$

where |g| is the absolute value of the determinant of the metric tensor, $r=r_{\Sigma }$ is the equation of the boundary surface of the source, and $\mu^{\left(L\right)}$ is the energy–density of the radiation associated exclusively with the dissipative processes related to the emission of gravitational radiation.

The above expression may be transformed further using a central result by Bondi ref. [22], relating the rate at which the energy is being radiated, with the news function, which reads:

(22) $$\frac{dm(u)}{du}=-\frac{1}{2}\int_{0}^{\pi }\frac{{\left(dc\left(u,\theta \right)\right)}^{2}}{du}\sin\theta d\theta ,$$

where $\frac{dc(u,\theta )}{du}$ is the news function, *u* is the timelike coordinate in the Bondi frame, c(u,θ) is a function entering into the power series expressions of the Bondi metric, and m(u) denotes the energy of the system (the Bondi mass).

Therefore, the total radiated energy in the timelike interval $u_{1}\leq u\leq u_{2}$ is given by

(23) $$E_{rad}^{\left(L\right)}=\int_{u_{1}}^{u_{2}}\left[\frac{1}{2}\int_{0}^{\pi }\frac{{\left(dc\left(u,\theta \right)\right)}^{2}}{du}\sin\theta d\theta \right]du,$$

(please notice a misprint in the sign of Equations (31) and (32) in ref. [26]).

On the other hand, according to the Landauer principle Equation (19), we obtain for the total number *N* of bits erased (radiated) in the process of the emission of gravitational radiation

(24) $$N=\frac{E_{rad}^{\left(L\right)}}{kT\sqrt{|g_{tt}|}\ln2},$$

Feeding back Equation (23) into Equation (24) we find an explicit relationship linking the news function with the total number of bits radiated in the assumed time interval,

(25) $$N=\frac{\int_{u_{1}}^{u_{2}}\left[\frac{1}{2}\int_{0}^{\pi }\frac{{\left(dc\left(u,\theta \right)\right)}^{2}}{du}\sin\theta d\theta \right]du.}{kT\sqrt{|g_{tt}|}\ln2}=\frac{\left[\int_{r_{\Sigma }}^{\infty }\int_{0}^{\pi }\int_{0}^{2\pi }\sqrt{\left|g\right|}\mu_{rad}^{\left(L\right)}drd\theta d\phi \right]}{kT\sqrt{|g_{tt}|}\ln2},$$

which measure the total erased information during the radiation process.

The expressions above have been obtained by resorting to the Landauer principle based on the Gibbs entropy; therefore, in the context of this work it is legitimate to ask how do the expressions above change if we use the Landauer principle based in the Tsallis entropy Equation (6). Using Equation (19) and retracing the same steps leading to Equation (25), we obtain

(26) $$N=\frac{\left(q-1\right)\int_{u_{1}}^{u_{2}}\left[\frac{1}{2}\int_{0}^{\pi }\frac{{\left(dc\left(u,\theta \right)\right)}^{2}}{du}\sin\theta d\theta \right]du.}{kT\sqrt{|g_{tt}|}\left[1-2^{\left(1-q\right)}\right]}=\frac{\left[\int_{r_{\Sigma }}^{\infty }\int_{0}^{\pi }\int_{0}^{2\pi }\sqrt{\left|g\right|}\mu_{rad}^{\left(L\right)}drd\theta d\phi \right]\left(q-1\right)}{kT\sqrt{|g_{tt}|}\left[1-2^{\left(1-q\right)}\right]},$$

bringing out the role played by the parameter *q* in the number of bits radiated in a given burst of gravitational radiation.

It would be most desirable to relate the above expressions with the data obtained from the LISA program (see ref. [27] and references therein). Unfortunately, at this point we do not see how to exactly establish such a link.

## 7. Discussion

Motivated by the fact that the specific value of the lower bound of energy—which according Landauer principle should be dissipated in the erasure of one bit of information—depends on the definition of entropy, we have addressed the question about the value of this bound for the Tsallis entropy, obtaining the expression Equation (10).

Once this value has been established, we have considered how deviations of this value, with respect to the one obtained from the Gibbs entropy, affects different scenarios where the Landauer principle is involved. In particular we have brought out how different values of *q* modify the values of different observational variables.

The first important result resides in the expression for the lower bound of energy dissipated after the erasure of one bit of information for the Tsallis entropy, which is now given by Equation (10). Figure 1 shows that for any value of temperature, such dissipated energy is a monotonically decreasing function of *q*, which is larger than the corresponding value for the Gibbs entropy for any value of *q* in the interval [0,1] and smaller in the interval [1,∞].

Next, we have considered the mass associated with a bit of information, which for the Tsallis entropy is given by Equation (14) in contrast with expression Equation (11) obtained from the Gibbs entropy. This result also affects the limitation on the speed of processing, as expressed by Equation (15).

The generalization of the Landauer principle for systems embedded in a gravitational field has been achieved following the work by Daffertshoffer and Plastino ref. [17]. The corresponding expression for the energy dissipated in the erasure of one bit of information is now given by Equation (19), leading to the expression Equation (20). Once again we see how *q* affects the values of the two above mentioned variables.

Finally, we addressed the question about the number of bits radiated (erased) in the emission of gravitational radiation. By using the Tsallis entropy, we found that such a number is given by Equation (26) instead of the expression Equation (25) corresponding to the Gibbs entropy.

In all these examples the role of the parameter *q* is clearly displayed. This fact brings us back to the leitmotiv of our work.

Indeed, it is to be expected that for any physical scenario, the experimental data could differentiate between what is the correct definition of entropy that should be adopted. In the case of Tsallis entropy, this implies a specific value of *q*. Since the scenarios analyzed above imply observed quantities, we harbor the hope that some of the expressions found here could help in a process of verification of the appropriate definition of entropy. Moreover, we believe that the extension of the program followed in this work to other definitions of entropy is an issue that deserves to be considered in the future.

## Data Availability

Data are contained within the article.
